# The distribution of potential West Nile virus vectors, *Culex pipiens pipiens *and *Culex pipiens quinquefasciatus *(Diptera: *Culicidae*), in Mexico City

**DOI:** 10.1186/1756-3305-4-70

**Published:** 2011-05-09

**Authors:** Alvaro Diaz-Badillo, Bethany G Bolling, Gerardo Perez-Ramirez, Chester G Moore, Jorge P Martinez-Munoz, America A Padilla-Viveros, Minerva Camacho-Nuez, Alfonso Diaz-Perez, Barry J Beaty, Maria de Lourdes Munoz

**Affiliations:** 1 Department of Genetics and Molecular Biology, Centro de Investigación y de Estudios Avanzados del Instituto Politécnico Nacional, Ave. Instituto Politécnico Nacional No. 2508, San Pedro Zacatenco-Gustavo A. Madero, México D.F., C.P. 07360, México; 2 Department of Microbiology, Immunology & Pathology, Colorado State University (CSU), Fort Collins, Colorado, USA; 3 Public Health Laboratory of State of Oaxaca, Km. 18.5 Carr. Oaxaca-Sola de Vega, Reyes Mantecon-Oaxaca de Juárez, C.P. 68000, Oaxaca México; 4 University of Tecamac, Tecamac-Estado de México, México; 5 Genomics Science Program, Autonomous University of México City, Ave. San Lorenzo No. 290, Del Valle-Benito Juárez, C.P. 03100, México; 6 Campamento de Obras Viales, Departamento del Distrito Federal, México D.F., México

## Abstract

**Background:**

*Culex *spp. mosquitoes are considered to be the most important vectors of West Nile virus (WNV) detected in at least 34 species of mosquitoes in the United States. In North America, *Culex pipiens pipiens, Culex pipiens quinquefasciatus*, and *Culex tarsalis *are all competent vectors of WNV, which is considered to be enzootic in the United States and has also been detected in equines and birds in many states of Mexico and in humans in Nuevo Leon. There is potential for WNV to be introduced into Mexico City by various means including infected mosquitoes on airplanes, migrating birds, ground transportation and infected humans. Little is known of the geographic distribution of *Culex pipiens *complex mosquitoes and hybrids in Mexico City. *Culex pipiens pipiens *preferentially feed on avian hosts; *Culex pipiens quinquefasciatus *have historically been considered to prefer mammalian hosts; and hybrids of these two species could theoretically serve as bridge vectors to transmit WNV from avian hosts to humans and other mammalian hosts. In order to address the potential of WNV being introduced into Mexico City, we have determined the identity and spatial distribution of *Culex pipiens *complex mosquitoes and their hybrids.

**Results:**

Mosquito larvae collected from 103 sites throughout Mexico City during 2004-2005 were identified as *Culex, Culiseta *or *Ochlerotatus *by morphological analysis. Within the genus *Culex*, specimens were further identified as *Culex tarsalis *or as belonging to the *Culex pipiens *complex. Members of the *Culex pipiens *complex were separated by measuring the ratio of the dorsal and ventral arms (DV/D ratio) of the male genitalia and also by using diagnostic primers designed for the *Ace.2 *gene. *Culex pipiens quinquefasciatus *was the most abundant form collected.

**Conclusions:**

Important WNV vectors species, *Cx. p. pipiens*, *Cx. p. quinquefasciatus *and *Cx. tarsalis*, are all present in Mexico City. Hybrids of *Cx. p. pipiens *and *Cx. p. quinquefasciatus *were also collected and identified. The presence and abundance of these WNV competent vectors is a cause for concern. Understanding the distribution of these vectors can help improve viral surveillance activities and mosquito control efforts in Mexico City.

## Background

Arthropod-borne viral (arboviral) infections are a major public health concern, causing considerable morbidity and mortality in humans and livestock throughout the world. There are more than 100 arboviruses that cause disease in humans, including members of the *Flaviviridae*, *Bunyaviridae*, and *Togaviridae *families [1]. Arboviral infections produce a broad spectrum of disease, ranging from asymptomatic infection to mild febrile illness or more severe conditions, such as encephalitis or hemorrhagic fever, which may result in long-term sequelae or death [2,3]. Human and animal pathogenic arboviruses such as West Nile virus (WNV), Chikungunya virus (CHIKV), Rift Valley fever virus (RVFV) and Bluetongue virus (BTV) have emerged and caused epidemics in North America, Europe and the Arabian Peninsula [1]. *Culex *are important vectors of West Nile virus and other arboviruses [2,3] in North America, and consequently prediction and monitoring of their abundance are central to arboviral surveillance and control programs.

West Nile virus (WNV) was first isolated in America from *Culex *mosquitoes and birds in New York City in 1999. Subsequently, the virus has spread westward across the country with the total numbers of reported cases exceeding 30,000 and more than 1200 fatalities occurring in the past 12 years [4]. WNV is maintained in nature by a bird-mosquito transmission cycle [5,6]. The most important mosquito vectors belong to the genus *Culex*, a very closely related group of mosquitoes originating in Africa [7,8]. *Culex *spp. mosquitoes are widespread and can be found in tropical and temperate climate zones on all continents except Antarctica [9].

*Cx. p. pipiens*, *Cx. p. quinquefasciatus*, and *Cx. tarsalis *are considered to be the primary vectors of WNV in North America for several reasons. First, they are the most common mosquitoes in urban areas [10-14]; WNV outbreaks typically occur during the peak abundance period of these vector species [15]; they are competent laboratory vectors of WNV [16]; and they have repeatedly been found infected with WNV in nature in the United States [17]. Unlike most other arboviruses, WNV has been detected in several genera and numerous species of mosquitoes, including 60 North American species and over 75 species from more than 10 genera worldwide [18]. *Culex pipiens *complex mosquitoes also serve as important vectors of St Louis encephalitis [19], Rift Valley fever [20] and Japanese encephalitis viruses [21].

The global distribution of the *Cx. p. pipiens*, *Cx. p. quinquefasciatus *and the North American distribution of *Cx. tarsalis *poses a threat for introduction and transmission of WNV into Mexico including Mexico City. *Culex *spp. mosquitoes are frequently detected on airplanes [22,23], and the international airport in Mexico City receives numerous flights from WNV endemic areas daily. WNV was introduced into Northern Mexico from the central United States in 2002, presumably via migration of viremic birds [24]. Either of these mechanisms could result in the introduction of WNV into the metropolitan area of Mexico City. The presence of a susceptible human population, approximately 20 million people, and abundant *Cx. pipiens *complex mosquitoes is of great public health concern.

The *Cx. pipiens *complex mosquitoes in Europe differ in behavior and physiology compare to the American mosquitoes and there is little evidence of gene flow between species [16]. *Cx. p. pipiens *have been implicated in urban outbreaks of WNV in Europe; however these outbreaks were nonrecurring and localized [16], whereas in the United States WNV is enzootic and widespread outbreaks occur. Interestingly, hybrids between *Cx. p. pipiens *and *Cx. p. quinquefasciatus *are widely found in the United States [16]. These hybrids, which presumably feed on birds as well as humans, may contribute to the sustained transmission of WNV to humans and horses in North America.

Because of their potential importance in serving as bridge vectors for WNV from avians to humans, we were interested in determining the frequency and distribution of *Cx. p. pipiens-Cx. p. quinquefasciatus *hybrids in Mexico City.

The *Cx. pipiens *complex is considered a controversial topic in mosquito taxonomy [25], because divergent physiological and behavioral traits occur without distinctive morphological differentiation. Two methods used to distinguish between *Cx. p. pipiens *and *Cx. p. quinquefasciatus *are: 1) DV/D ratio, and 2) PCR amplification of acetylcholinesterase (Ace.2*) *gene sequences. The DV/D ratio refers to the relative overlap and measurement of the dorsal and ventral arms in male genitalia. DV/D ratios for *Cx. p. pipiens *are less than 0.2, for *Cx. p. quinquefasciatus *they are greater than 0.4, and hybrids have intermediate ratios between 0.2-0.4 [26,27]. PCR amplification of Ace.2 gene sequences result in amplicons of different sizes specific for the two taxa [28-30].

In this study, mosquitoes were differentiated by both the male DV/D ratio measurements and by the Ace.2 gene PCR product analysis [30]. Mosquito larvae were collected from breeding sites, reared to adults, and DV/D ratios were determined in males. Morphological and molecular results were used to determine whether the mosquito was *Cx. p. quinquefasciatus*, *Cx. p. pipiens *or a hybrid between the two species.

Determining the distribution of *Cx. p. pipiens, Cx. p. quinquefasciatus*, their hybrids and *Cx. tarsalis *is necessary to determine the epidemic potential of WNV in Mexico City. This information will provide base-line information for initiating surveillance programs and initiating control activities in the event that WNV is introduced into the region.

## Methods

### Study area

Mexico City, the capital city of Mexico, is a Federal District. The Distrito Federal is at the same administrative level as the states. It is located in the Valley of Mexico, also called the Basin of Mexico or the Valley of Anáhuac, a large valley located in the Trans-Mexican Volcanic Belt in the high plateaus at the center of Mexico. It has a minimum elevation of 2,200 meters above sea level and is surrounded by mountains and volcanoes that reach elevations of over 5,000 meters. The city primarily rests on what was once Lake Texcoco. The entire lakebed is now paved over and most of the city's remaining forested areas lie in the southern boroughs of Milpa Alta, Tlalpan and Xochimilco. The city has a temperate highland climate [31], due to its tropical location and high elevation. The lower region of the valley receives less rainfall than the higher regions of the south. The lower boroughs of Iztapalapa, Iztacalco, Venustiano Carranza and the west portion of Gustavo A. Madero are usually drier and warmer than the upper southern boroughs of Tlalpan and Milpa Alta, a mountainous region of pine and oak trees known as the range of Ajusco, Tlalpan. Seasonally, the lowest temperatures usually register during January and February, reaching -2 to -5°C (28 to 23°F), sometimes accompanied by snow showers on the southern regions of Ajusco. The maximum temperatures occur during late spring and summer reaching up to 32°C (90°F). The area receives about 700 mm of annual rainfall, which is concentrated from June through September/October with little or no precipitation the remainder of the year. Mexico City is a prominent economic, industrial, and cultural center in the country and is the most populous city with over 8,836,045 inhabitants in 2008 [32]. The Federal District is divided into 16 districts or boroughs. The boroughs are composed of hundreds of neighborhoods [33]. The metropolitan area, Zona metropolitana del Valle de México, consists of the Federal District and 60 other municipalities, mainly located north and east of Mexico City. This area has a total population of approximately 20,000,000 inhabitants (2010 estimate, 29).

### Mosquito collection and identification

**Figure 1:**
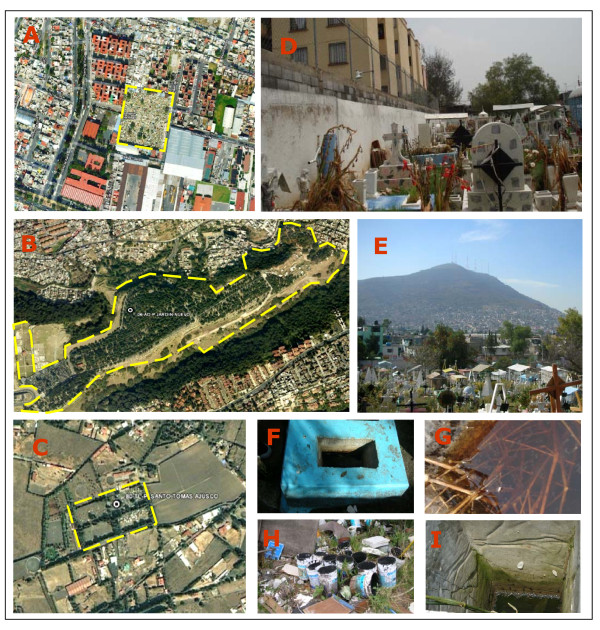
**Landscape and habitats of mosquito larval collection sites**. (A) Urban area, representative urban area in cemeteries of Mexico City characterized by high human population density, public transportation and communication containing a small amount of green areas. (B) Suburban, this area is constituted for a balance between buildings and green areas with sufficient public transportation and communication but where the people are not dedicated to agricultural activities. (C) Rural, this representative area is characterized by low density human population, with little public transportation and communication and extensive green and agricultural areas. (D-I) Display all varieties of natural and man-made containers representative of the mosquito larval habitats in the collection sites at the cemeteries.

Mosquitoes were collected as larvae aboveground from various urban (Figure 1A), suburban (Figure 1B) and rural cemeteries (Figure 1C) in Mexico City during 2004-2005. This represents the first study describing mosquito larval habitats in Mexico City. Larvae were collected from tree holes, ditches, decorative ponds, flower pots, buckets, and water-retaining debris from each cemetery (Figure 1D-I). The water in the collection sites was present for different periods of time. Temporal analysis of mosquito population dynamics was addressed by performing monthly collections on six occasions from May through October 2005. Larvae were carried alive to the insectary where they were reared to adults and examined after emergence. Larvae were reared under standard conditions and pupae were placed (approximately 200 pupae/cup) in cages (30 × 30 × 30 cm) for adult emergence (approximately 400 adults/cage). Mosquitoes were maintained at 28°C ± 2°C and photoperiod of 14:10 (L:D) h.

Specimens were identified to species with the aid of a stereomicroscope by using standard identification keys and recently described characters [34] including the DV/D ratio descriptive key [35]. *Culex pipiens *complex specimens were further subjected to a species-specific polymerase chain reaction (PCR) test based on ribosomal DNA (Ace.2 gene) to confirm the results of morphologic identifications [30,34]. Identified specimens were either processed immediately for genomic DNA extraction or stored at -80°C.

### Genomic DNA extraction

Genomic DNA extraction was performed on individual male mosquitoes. Each mosquito was homogenized with the aid of a microtube pestle (USA Scientific, Enfield, CT) in a 1.5 ml tube containing 180 μl phosphate buffered saline (PBS, 137 mM NaCl, 2.7 mM KCl, 4.3 mM Na2HPO4, 1.47 mM KH2PO4) and subjected to DNA extraction according to Garcia-Franco, et al. [36]. Isolated DNA from each mosquito was reconstituted in 50 μl Tris-EDTA (TE) buffer (10 mM Tris-Cl, 1 mM EDTA, pH 8.0), and stored at -20°C for PCR [28,30].

### Polymerase chain reaction assay

The PCR reaction was carried out by incubation of 0.20 μM of the corresponding sense and antisense PCR primers, 1× PCR buffer, 250 μM of each dNTP, 2 mM MgCl_2_, 0.15 mg/ml of bovine serum albumin, 2.5 units of Taq polymerase (Applied Biosystems), and approximately 6 ng of genomic DNA per the manufacturer's recommendations.

Amplification of the Ace.2 gene was performed using primers:

ACEquin (5'-CCTTCTTGAATGGCTGTGGCA-3'),

ACEpip (5'-GGAAACAACGACGTATGTACT-3') and

B1246 (5'-TGGAGCCTCCTCTTCACGGC-3') described previously [30]. The PCR conditions were as described previously, briefly: one cycle at 94°C for 5 min, followed by 35 cycles of 94°C for 30 seconds, 56°C for 30 seconds, 72°C for 60 seconds, and one final extension of 72°C for 5 min.

## Results

### Species of collected mosquitoes

**Figure 2:**
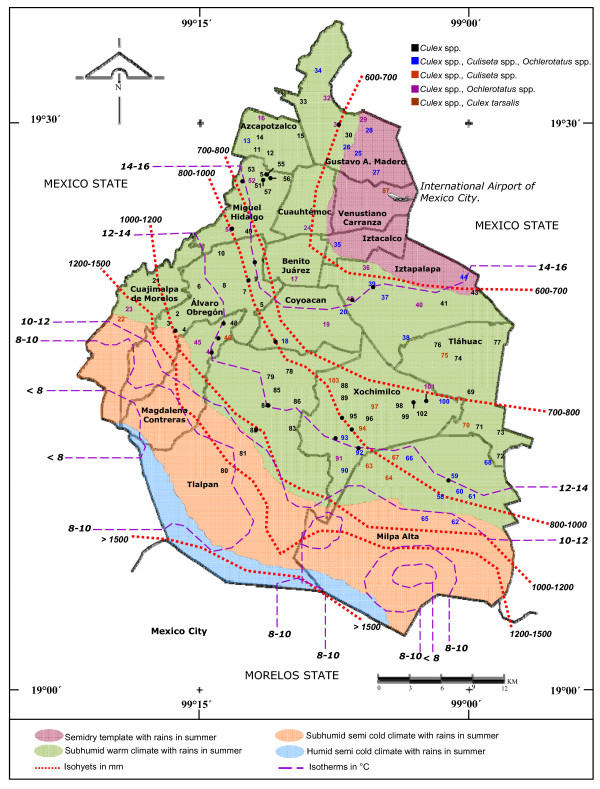
**Location of mosquito sampling sites and species detected in Mexico City for 2004**. The map of Mexico City displays the distribution of the mosquito species identified in the collection sites with some climatic and geographic features including humidity, isohyets, isotherms and surrounding states. Numbers in black indicate morphological identification of *Culex *spp.; in blue *Culex *spp., *Culiseta *and *Ochlerotatus; *in red *Culex *spp. and *Culiseta; *in purple *Culex *spp. and *Ochlerotatus; *and in brown *Culex *spp. and *Cx. tarsalis*.

**Figure 3:**
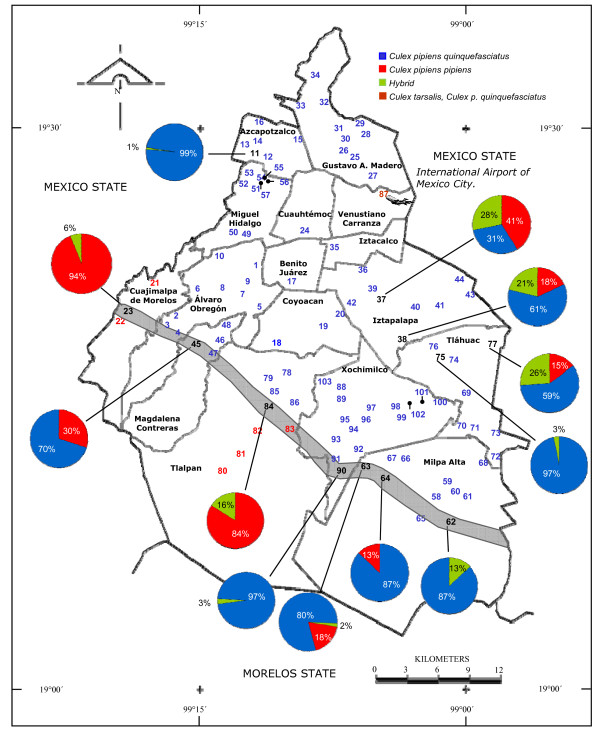
***Culex pipiens *complex distribution in Mexico City**. Map of *Cx. p. quinquefasciatus*, *Cx. p. pipiens *and hybrid distribution in all collection sites according to nucleotide differences in the Ace.2 gene. Pie chart graphs (numbers in black) indicate the distributional frequency of identified lineages. The sizes of the pie chart's segments are proportional to the number of mosquitoes identified as *Cx. p. quinquefasciatus *(blue), *Cx. p. pipiens *(red) or hybrid (green). The numbers in blue indicate the sites where *Cx. p. quinquefasciatus *was exclusively identified, red where *Cx. p. pipiens *was recognized and in black where hybrids were localized according to chart graphs. In addition the number in brown indicates the site where *Cx. p. quinquefasciatus *and *Cx. tarsalis *was observed. The grey line approximates the probable hypothetical introgression area. The numbers correspond to the collection sites displayed in the Additional File 1.

A total of 202,148 mosquito larvae belonging to the genera *Culex *(77%), *Culiseta *(18%) and *Ochlerotatus *(5%) were collected from 3,955 containers from cemeteries in 16 Districts in Mexico City (Additional file 1). Larvae were collected in all sites; 49.5% of the collected sites yielded *Cx. pipiens *complex mosquitoes exclusively; 40.7% of the sites yielded *Ochlerotatus *mosquitoes, and 33.9% of the sites yielded *Culiseta *mosquitoes. Interestingly, *Cx. tarsalis *was identified only in Peñon de los Baños (site 87-VC) close to the international airport (Figure 2, 3) coexisting with *Cx. p. quinquefasciatus*. Furthermore, *Cx. pipiens *complex were coexisting with *Culiseta *and *Ochlerotatus *in 25 sites, with *Culiseta *in 10 sites, and with *Ochlerotatus *in 17 sites (Figure 2). In this study, we used 3 different land cover classes, urban, suburban and rural areas (Figure 1A-C) to characterize the landscape in different regions of Mexico City. The locations of all mosquitoes belonging to the different genera are displayed in Figure 2.

Out of 10,250 *Cx. pipiens *complex mosquitoes examined, 54.5% were females and 45.5% were males (Additional file 1). The male to female ratio was 0.84:1.

### Identification of *Culex pipiens *complex members in Mexico City by DV/D analysis

The locations of the *Culex *complex mosquito collection sites are shown in Figure 3. The mean value of DV/D ratio of the specimens examined in this study was used to distinguish *Cx. p. pipiens *from *Cx. p. quinquefasciatus *(Table 1). *Cx. p. pipiens *had DV/D ratios of less than 0.2, *Cx. p. quinquefasciatus *had DV/D ratios greater than 0.4 and hybrids displayed intermediate ratios of 0.2-0.4 (Table 1). Most collections consisted of *Cx. p. quinquefasciatus *(73.8%) alone and five collections contained only *Cx. p. pipiens *(4.3%). Two collections consisted of a combination of *Cx. p. pipiens *and *Cx. p. quinquefasciatus *(1.9%); *Cx. p. pipiens, Cx. p. quinquefasciatus *and hybrids (7.8%); *Cx. p. pipiens *and hybrids (1.9%) or *Cx. p. quinquefasciatus *and hybrids (9.7%). Figure 3 shows the locations where *Cx. p. quinquefasciatus *(numbers in blue) or *Cx. p. pipiens *(numbers in red) were identified by morphological analysis and confirmed by molecular analyses (Ace.2 gene).

**Table 1:** Climatic conditions and percentage of hybrids collected from the different cemeteries.

Key	Rain(mm/year)	^!^T (°C)	^†^W. T. (°C)	^&^R. H. (%)	pH	Total larvae	Morphological Identification (%)			Molecular Identification (%)		
							***Cx. p.*** *** quinque-*** ***fasciatus***	***Cx. p.*** *** pipiens ***	**Hybrid**	***Cx. p.*** *** quinque-*** ***fasciatus***	***Cx. p. *** ***pipiens ***	**Hybrid**

**AO**	**29.1**	**15.1**	**14.2**	**33.8**	**7.2**							

03-AO**	38	15.4	13.9	36	7.9	2030	91	0	9	100	0	0

**AZ**	**69.8**	**17.5**	**15.5**	**39.3**	**6.9**							

11-AZ*	61	17	15	42	7	2200	98	0	2	99	0	1

**CJ**	**225**	**13.3**	**11**	**43**	**7.1**							

21-CJ**	225	14	13	56	7.2	2790	0	81	19	0	100	0

22-CJ***	225	11	8	37	7	1360	0	100		0	100	0

23-CJ**	225	15	12	36	7.1	865	0	72	28	0	94	6

**GM**	**105.7**	**18**	**17.2**	**47.8**	**7.3**							

26-GM*	107	17	15	56	7.2	710	98	0	2	100	0	0

**IP**	**90.1**	**17.1**	**15.6**	**40.1**	**7.2**							

37-IP*	85	16	14	57	7.2	4765	22	39	39	31	41	28

38-IP***	85	17	15	52	7.5	2345	53	22	25	61	18	21

39-IP*	85	15.5	14	34	7.2	1710	99	1	0	100	0	0

44-IP*	94	16	16	36	7	2640	97	0	3	100	0	0

**MC**	**204**	**12.5**	**11.5**	**57.5**	**7.18**							

45-MC*	204	12	11	62	7.3	845	67	29	4	70	30	0

47-MC*	204	12	10	58	7.1	995	67	0	33	100	0	0

**MA**	**140**	**14.2**	**13.4**	**34.7**	**7.4**							

62-MA**	140	13.8	12.5	34	7.3	2895	73	0	27	87	0	13

63-MA***	140	13.2	11.8	36	7.7	1845	68	9	23	80	18	2

64-MA***	140	13.6	12.2	35	7.8	2420	87	13	0	87	13	0

65-MA**	140	13.9	13	33	6.9	619	94	0	6	100	0	0

**TH**	**111.2**	**16.2**	**15.9**	**59.1**	**7**							

73-TH*	107	16	16	82	7.2	2680	71	0	29	100	0	0

75-TH**	156	16	15.5	62	6.9	1140	56	3	41	97	0	3

77-TH***	96	17	16.5	52	6.6	1290	61	18	21	59	15	26

**TL**	**184.1**	**12.3**	**9.8**	**44.9**	**7.2**							

78-TL*	145	13.5	11	35	7	3720	82	0	18	100	0	0

80-TL***	219	8	6	55	7.8	1450	0	100	0	0	100	0

81-TL***	219	11.5	7	52	7.3	1795	0	100	0	0	100	0

82-TL**	219	12.5	7	54	7.1	2130	0	100	0	0	100	0

83-TL**	219	11	8	52	7.2	1690	0	100	0	0	100	0

84-TL**	145	13	11.5	42	7.2	940	12	37	51	0	84	16

**XOo**	**78.8**	**15.7**	**13.9**	**36.3**	**7.1**							

90-XO***	177	9	5	27	7.1	930	88	2	10	97	0	3

91-XO**	116	8	8	28	7	2590	76	0	24	100	0	0

^!^Temperature, ^†^Water temperature, ^&^Relative humidity, *Urban, **Suburban, ***Rural.

### Molecular Analyses

**Figure 4:**
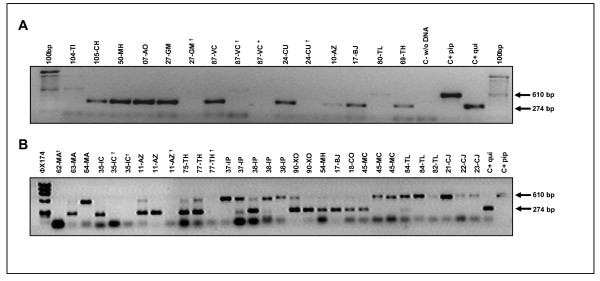
**PCR amplification of the Ace.2 gene**. Larvae collected at various cemeteries in Mexico City were reared to mosquito adults and then identified by PCR amplification of the Ace.2 gene [30]. The *Cx. p. pipiens *specific band of 610 bp and *Cx. p. quinquefasciatus *specific band of 274 bp and the expected bands of 610 and 274 bp for hybrids (panel B) are displayed in the agarose gels. *Cx. tarsalis identified by morphological analysis did not produce a PCR amplicon. ^†^*Culex spp*. mosquitoes that were not identified as pertaining to the *Cx. pipiens *complex or hybrids by morphological analysis or by the Ace.2 gene assay.

In order to distinguish the two nominal taxa, the Ace.2 gene was amplified by polymerase chain reaction (PCR) to detect taxa-specific amplicons. The representative results of the Ace.2 PCR assay for mosquitoes collected from cemeteries in Mexico City are shown in Figure 4. The PCR products of 610 and 274 bp were observed for *Cx. p. pipiens *and *Cx. p. quinquefasciatus *respectively and both fragments were detected for hybrids (Figure 4-B). The mosquitoes morphologically identified as *Cx. tarsalis *did not yield any PCR product as expected (Figure 4-A, line 10). The DV/D results were generally concordant with the results from the molecular (Ace.2 gene amplification) results. Out of all mosquitoes analyzed there were only 9 mosquitoes identified as hybrids by DV/D analyses that were identified as *Cx. p. quinquefasciatus *by molecular analysis in (03-AO) Santa Rosa Axochiac (Table 1). In areas where there may be introgression (Figure 3, faint gray line), individuals with *Cx. p. pipiens *DV/D ratios were identified as hybrids by molecular analysis and a few with *Cx. p. quinquefasciatus *DV/D ratios were also identified as hybrids. Interestingly, the most "observable" band correlated best with the morphological analysis. Our molecular analysis revealed that the frequency of members of the *Cx. pipiens *complex in the collection sites was: *Cx. p. quinquefasciatus *= 95.5%, *Cx. p. pipiens *= 14% and hybrids = 10%.

*Cx. p. pipiens *or *Cx. p. quinquefasciatus *were identified during the whole year in El Calvario (21-CJ), Cuajimalpa de Morelos and in Sanctorum (52-MH), Miguel Hidalgo respectively (Additional file 2). Furthermore hybrids identified in the samples from San Nicolas Tolentino in Ixtapalapa (37-IP) and from La Concordia in Cuajimalpa (23-CJ) are displayed in the Additional file 2.

### Seasonal Rainfall Contribution

In order to determine the influence of seasonal rainfall on the abundance of *Cx. pipiens *complex-mosquitoes, we analyzed molecularly 600 mosquitoes/month collected in six cemeteries during the rainy season from May to October in 2005 (Table 2). *Culex pipiens-quinquefasciatus *hybrid mosquitoes were found in all six collection sites. Mosquitoes were collected once per month from 10 different containers at each site. Hybrids were detected in the Azcapotzalco site from Jun to October, in the Milpa Alta site in July, in the Cuajimalpa site from July to October, in the Tlahuac site from June to October, in the Ixtapalapa site in June and from August to October, and finally in the Xochimilco site in June and July (Table 2). Furthermore, hybrid mosquito densities were qualitatively higher during the rainy season. Hybrids were not observed throughout the year. Notably they were not detected in the dry months (January to May). Two peaks in abundance of hybrid mosquitoes were observed (Additional file 2): the first in June and the second in August at the cemetery Civil San Nicolas Tolentino (37-IP site). Temperatures at the collection sites fluctuated from 8°C to 23°C. Hybrids were found at temperatures from 12.5°C to 17°C.

**Table 2:** Members of the *Culex pipiens *complex and hybrids identified by Ace

Cemetery	MAY	JUN	JUL	AUG	SEP	OCT
Santa Lucia, Azcapozalco	q	**h**	**h**	**h**	**h**	**h**

La Concordia, Cuajimalpa	q	p	**h**	**h**	**h**	**h**

San Nicolas Tolentino, Iztapalapa	q	**h**	p	**h**	**h**	**h**

San Salvador Cuauhtenco, Milpa Alta	ND	q	**h**	p	p	p

Santa Catarina, Tlahuac	q	**h**	**h**	**h**	**h**	**h**

San Francisco Tlalnepantla, Xochimilco	p	**h**	**h**	q	q	p

Samples were taken from six cemeteries and tested for Ace.2 gen to determine *Cx. p. quinquefasciatus *(q), *Cx. p. pipiens *(p) or their hybrids (h).

## Discussion

The mosquitoes belonging to the genera *Culex, Culiseta *and *Ochlerotatus *collected and identified in Mexico City are capable of transmitting pathogens causing many different diseases, including WNV. Mosquitoes belonging to the *Cx. pipiens *complex, their hybrids, and *Cx. tarsalis *are considered important vectors for WNV, which can cause fever, death, and long term sequelae in infected humans and also death in horses [37]. WNV is primarily enzootic among birds [38], with humans and equines serving as incidental or dead-end hosts [39]. Migratory birds can spread the virus over long distances [40,41]. WNV is responsible for human outbreaks in the United States, Europe, and the Middle East [6,15,42]. Mexico City has similar climatic conditions to other cities where outbreaks of WNV have occurred. West Nile virus has been reported in six Mexican states [43,44], which increases the probability of introduction of the virus into Mexico City. Little is known about the distribution of *Cx. pipiens *complex mosquitoes in the city. This information is essential for assessing the epidemic potential of WNV in Mexico City. West Nile Virus has been detected in *Culex *spp. mosquitoes in the United States since 1999, and has spread across most states [42], resulting in 30,658 human cases with 1,206 deaths by the end of 2010. The virus reached Canada in 2001 and countries of the Caribbean and Central America by 2003 [44,45]. WNV was detected in horses in the Mexican states of Veracruz, Yucatan, Chihuahua, Coahuila, Tamaulipas, Tabasco in 2002 [24]. However, the virus was not detected in horse samples from Durango, San Luis Potosí, Jalisco, Distrito Federal, Guerrero, Puebla, Oaxaca and Chiapas were negative for WNV [24]. The first two autochthonous human cases of confirmed WNV infection in Mexico were reported in Nuevo Leon and Sonora in 2005 [45]. To our knowledge, only 8 human cases of WNV infection have occurred in Mexico, 3 of which were severe but did not result in death [41]. One fatal human case was reported in Nuevo Leon, Mexico in 2005 [44] and 6 more cases in the northern Mexico in a survey from 2005 to 2007 [46]. In addition there are other reports that suggest that because there are many cases of dengue (DENV) in Mexico it also may be that the human Mexican hosts had antibodies against DENV that can block WNV activity [46]. However, since there has been no DENV activity in Mexico City, people from Mexico City could more likely be infected by the virus, if antibodies against DENV have any role in blocking WNV infection. The introduction and establishment of WNV in Mexico City--which would be a public health disaster--is a possibility because the *Culex *vectors are present throughout the city.

Virus introduction could occur in a variety of ways including migration of WNV infected birds or humans or by infected mosquitoes on airplanes. WNV outbreaks that occur sporadically in southern Europe are attributed to importation of the virus by migratory birds from Africa, which then infect local mosquitoes [47]. *Culex *spp. mosquitoes are frequently collected from airplanes [48], and it is possible that WNV could have been introduced into New York City in 1999 by an infected mosquito transported from the Middle East. In this regard Mexico City is served by Mexico City International Airport, Latin America's busiest and largest airport, with regular (daily) flights to North America, mainland Mexico, Central America and the Caribbean, South America, Europe and Asia, has code share agreements spanning the entire globe. It is now used by over 26 million passengers per year [49], and in 2008, about 31 million people went through the city's airports. With high traffic volume to and from endemic areas Mexico City is at risk for introduction of WNV. Interestingly, *Cx. tarsalis *was only detected in one site close to the international airport suggesting possible transport of this mosquito via aircraft as has occurred in other countries [48]. This suggestion is based on the fact that we did not find this mosquito in any other collection sites, even though collections were made in natural ground pools and water present for less than a week that is preferred by *Cx. tarsalis *[48]. Our results display for the first time the prevalence and distribution of the *Cx. pipiens *complex in the city, which will help to vector control efforts in the event of introduction of the virus. We note that in this study only larval habitats from cemeteries were sampled to make the most efficient use of available resources. A broader sample of land use classes (e.g., industrial, high-density housing, commercial, etc.) may have produced slightly different results. Similarly, the use of light traps would likely produce different relative abundances given the known differences in response to light traps. In the case of larval sampling versus light traps, larval sampling--if it is sufficiently thorough--is more likely to sample the entire complex of species. Cemeteries, because of their accessibility, are an effective proxy for the larger surrounding area. Surveys of other land use classes are planned in future projects.

It is very clear that *Cx. p. pipiens *and *Cx. p. quinquefasciatus *are closely associated with humans. Hybrid zones between the two species are known to occur in North America, Argentina, and Madagascar [25,50,51]. Mexico City has approximately 103 cemeteries surrounded by houses and apartments (Figure 1). The crypt vases contain water throughout the year, providing mosquito breeding sites [52]. Cemeteries sampled during this study confirmed the presence of *Cx. pipiens *complex and *Cx. tarsalis *mosquitoes in Mexico City. Our results show for the first time that *Cx. p. pipiens, Cx. p. quinquefasciatus*, their hybrids and *Cx. tarsalis *are all present in Mexico City. Previously, *Cx. p. pipiens *was thought to be found only in the United States and Canada [26]. Furthermore, Mexico City was not included in those previous reports.

Based upon DV/D values and ribosomal DNA analysis [30], specimens from extreme isotherms 12-14 °C to the North of Mexico City were unambiguously identified as *Cx. p. quinquefasciatus*. This is the first report for Mexico City and it is in agreement with previous studies showing that only *Cx. p. quinquefasciatus *is usually found south of 36° N in North America [26]. *Culex pipiens pipiens *mosquitoes were detected above isotherms 12-14 °C to the South of Mexico City. Finally, *Cx. p. pipiens-quinquefasciatus *hybrids were detected between isotherms 10-12 °C and isotherms 12-14°C at Cuajimalpa, Alvaro Obregon, Magdalena Contreras, Tlalpan, Xochimilco and Milpa Alta Districts (Figure 2). *Culex pipiens pipiens *mosquitoes were also collected during the whole year in the cemetery "El Calvario" in Cuajimalpa de Morelos, suggesting mosquito adaptation to this region.

Hybrids were collected in the rainy season from June to October from northwest to southeast of the city, where there was high mosquito density. The abundance of hybrid mosquitoes increased in the late summer (Cuajimalpa, Iztalapa, Azcapozalco, Xochimilco, Milpa Alta and Tlahuac) between September and October associated with a decrease in temperature (Table 2).

We are suggesting a hypothetical hybridization region (Figure 3) revealing the distribution limits of *Cx. p. quinquefasciatus *and *Cx. p. pipiens *without any apparent geographic barrier. This is based on the apparent spatial limits of *Cx. p. quinquefasciatus *and *Cx. p. pipiens *populations, with the localization of hybrid mosquitoes occurring between them. Additional studies are needed to demonstrate that this is a true introgression area. *Culex pipiens pipiens *were detected principally in suburban or rural lands and *Cx. p. quinquefasciatus *were detected primarily in urban zones, which has been reported previously [53]. Interestingly, in the Northeast districts of Iztapalapa and Tlahuac, there was no apparent division between the *Cx. pipiens *complex mosquitoes; with *Cx. p. quinquefasciatus*, *Cx. p. pipiens *and hybrids found in the same collection sites (Figure 3).

It has been suggested that hybridization between *Cx. p. pipiens *and *Cx. p. quinquefasciatus *mosquitoes may have facilitated the rapid spread of West Nile virus in North America [16]. Our studies support the presence of a self-sustaining hybrid population in Mexico City (Figure 2). This is similar to results of previous studies in Colorado [28], California [25,54], and Tennessee [55]. The nature of the hybrid populations in Mexico City requires further study. Microsatellite markers have been used to assess introgression between *Cx. p. pipiens *and *Cx. p. quinquefasciatus *[56], and this population genetic approach could be used to further define the extent and geographic regions of introgression. It should also be noted that the presence of *Cx. p. pipiens *itself could pose a risk to humans in Mexico City, as there are reports that this species may shift feeding preferences from birds to humans in urban areas during late summer and early fall [57].

Our analyses provide insight into the distribution of *Cx. pipiens *complex mosquitoes in Mexico City. This is important as behavioral differences, such as host-feeding preferences, are important in determining the vectorial capacity of mosquitoes from this complex. This information is necessary for establishing effective surveillance and targeted control programs to prevent or control WNV outbreaks.

## Conclusions

*Culex pipiens pipiens*, *Cx. p. quinquefasciatus *and *Cx. tarsalis *are important vectors for WNV, that occur in Mexico City. Hybrids of *Cx. p. pipiens *and *Cx. p. quinquefasciatus *were detected by DV/D ratio and molecular analysis. Hybrids were identified during the rainy season from June to October, in an introgression region extending from the northwest to southeast of the city. Clearly, Mexico City has an abundance of competent *Culex *spp. vectors and is at risk for introduction of WNV and epidemic disease. Increased surveillance and effective vector control should be implemented in order to avoid the risk of an epidemic of WNV in this highly populated city.

## Competing interests

The authors declare that they have no competing interests.

## Authors' contributions

ADB made all mosquito collections, carried out the morphological and molecular analysis of mosquitoes and participated in the writing and discussion of results; BGB and CGM supported molecular analysis confirmation, read and approved the final manuscript; GPR helped to rear the mosquitoes, participated in the writing and discussion of results and assisted with the literature validation; JPMM helped to confirm the morphological mosquito analysis of all collections; AAPV participated in the molecular analysis of the mosquitoes collected; MCN participated in discussion of results; ADP helped to make all mosquito collections; BJB and CGM participated in discussion of results, review of the manuscript, assisted with the literature selection and validation, and proof-read the manuscript; MLM participated in writing, analysis and discussion of results, review the manuscript, assisted with the literature validation and proof-read and assembled the manuscript. All authors participated in the discussion of results and read and approved the final manuscript.

## Supplementary Material

Additional File 1 **Geographic location of cemeteries and percentage of male mosquitoes obtained**. Click here for file

Additional File 2 **PCR amplification of the Ace.2 gene from two different cemeteries in 2005**. (A) Samples from cemetery "El Calvario" in Cuajimalpa de Morelos borough. All PCR products correspond with the *Cx. p. pipiens *(610 bp) in each month during 2005. B) Samples from cemetery "Sanctorum" in Miguel Hidalgo borough where the PCR products correspond with *Cx. p. quinquefasciatus *(274 bp) in each month during 2005. C) Samples from "San Nicolas Tolentino" in Ixtapalapa (37-IP) and from "La Concordia" in Cuajimalp (23-CJ) boroughs where the PCR products correspond with hibryds (610 and 274 bp. Negative controls are indicated as C-Cs.DNA (Culiseta). Positive controls are indicated as C+pip, C+qui and 52-MH2004(C+). 100 bp DNA Ladders are in lines 1 panel A, line 17 panel B and line 1 panel C on the right side; and φ X174 DNA/Hae III ladder on panel C left side. Arrows show the bands of 274 bp and 610 bp in all panels. Click here for file
