# Supplementary material for: The distribution of potential West Nile virus vectors, Culex pipiens pipiens and Culex pipiens quinquefasciatus (Diptera: Culicidae), in Mexico City
Source: Parasit Vectors. 2011 May 9;4:70. doi: 10.1186/1756-3305-4-70 (PMC3117809; doi:10.1186/1756-3305-4-70)
Supplement: Additional File 1 — Geographic location of cemeteries and percentage of male mosquitoes obtained. [file 1756-3305-4-70-S1.DOC]

### Additional File 1 – Geographic location of cemeteries and percentage of male mosquitoes obtained.

| **Key** | **Cemetery** | **Latitude** | **longitude** | **Altitude (m)** | **Date (2004)** | **Containers**  **(number)** | **Total Larvae** | **Male**  **(%)** |
| --- | --- | --- | --- | --- | --- | --- | --- | --- |
| **AO** | **Alvaro Obregon☼** |  |  |  |  |  |  |  |
| 01-AO | Guadalupe* | 19°22'47.12'' | 99°11'53.24'' | 2293 | 04/04 | 24 | 2680 | 28 |
| 02-AO | San Ramon** | 19°20'09.21'' | 99°16'43.71'' | 2694 | 03/04 | 14 | 1350 | 32 |
| 03-AO | Santa Rosa Axochiac** | 19°20'03.32'' | 99°16'49.79'' | 2697 | 03/04 | 28 | 2030 | 39 |
| 04-AO | Guadalupe Tlacoyaque** | 19°19'33.06'' | 99°16'09.52'' | 2698 | 03/04 | 12 | 1060 | 34 |
| 05-AO | San Rafael* | 19°20'13.69'' | 99°11'31.23'' | 2297 | 04/04 | 16 | 1100 | 32 |
| 06-A0 | Jardin-Nuevo** | 19°20'58.08'' | 99°15'28.00'' | 2259 | 03/04 | 28 | 1650 | 49 |
| 07-AO | Jardin* | 19°21'00.97'' | 99°13'04.34'' | 2413 | 03/04 | 72 | 6240 | 32 |
| 08-AO | Balcones De Cehuayo** | 19°21'24.35'' | 99°14'27.58'' | 2541 | 03/04 | 8 | 420 | 33 |
| 09-AO | Tarango* | 19°21'45.47'' | 99°12'30.23'' | 2330 | 04/04 | 22 | 1740 | 43 |
| 10-AO | Civil Santa Fe* | 19°23'03.41'' | 99°13'50.32'' | 2413 | 04/04 | 28 | 960 | 46 |
| **AZ** | **Azcapozalco☼** |  |  |  |  |  |  |  |
| 11-AZ | Santa Lucia* | 19°28'33.47'' | 99°11'57.30'' | 2257 | 01/05 | 25 | 2200 | 41 |
| 12-AZ | Santa Apolonia* | 19°28'25.04'' | 99°11'35.28'' | 2254 | 01/05 | 20 | 1350 | 33 |
| 13-AZ | San Isidro* | 19°29'12.91'' | 99°12'45.68'' | 2258 | 02/05 | 115 | 4235 | 39 |
| 14-AZ | San Juan Tlihuaca* | 19°29'15.77'' | 99°12'21.05'' | 2256 | 02/05 | 19 | 1620 | 40 |
| 15-AZ | Santa Cruz De Las Salinas* | 19°29'19.89'' | 99°09'18.06'' | 2245 | 04/09 | 16 | 1100 | 47 |
| 16-AZ | San Jose* | 19°30'02.55'' | 99°11'50.95'' | 2252 | 02/05 | 17 | 890 | 38 |
| **BJ** | **Benito Juarez☼** |  |  |  |  |  |  |  |
| 17-BJ | Panteon Xoco* | 19°21'33.97'' | 99°09'55.87'' | 2256 | 28/03 | 17 | 2130 | 42 |
| **CO** | **Coyoacan** |  |  |  |  |  |  |  |
| 18-CO | Mausoleos Del Angel* | 19°18'25.48'' | 99°10'44.25'' | 2302 | 29/05 | 14 | 710 | 31 |
| 19-CO | San Pablo Tepetlapa* | 19°19'19.33'' | 99°08'18.38'' | 2249 | 29/05 | 25 | 810 | 52 |
| 20-CO | San Francisco Culhuacan* | 19°20'02.69'' | 99°07'13.64'' | 2242 | 29/05 | 20 | 2220 | 43 |
| **CJ** | **Cuajimalpa☼** |  |  |  |  |  |  |  |
| 21-CJ | El Calvario** | 19°21'34.14'' | 99°18'02.07'' | 2792 | 05/06 | 42 | 2790 | 39 |
| 22-CJ | San Lorenzo*** | 19°19'53.48'' | 99°19'43.52'' | 2924 | 05/06 | 19 | 1360 | 48 |
| 23-CJ | La Concordia** | 19°21'04.33'' | 99°19'07.00'' | 2753 | 05/06 | 22 | 865 | 48 |
| **CU** | **Cuauhtemoc☼** |  |  |  |  |  |  |  |
| 24-CU | Frances* | 19°24'16.94'' | 99°09'14.00'' | 2241 | 28/03 | 32 | 1564 | 32 |
| **GM** | **Gustavo A. Madero☼** |  |  |  |  |  |  |  |
| 25-GM | Progreso* | 19°29'03.23'' | 99°06'37.33'' | 2237 | 03/07 | 132 | 5620 | 48 |
| 26-GM | Del Tepeyac* | 19°29'13.54'' | 99°06'56.71'' | 2270 | 03/07 | 36 | 710 | 43 |
| 27-GM | Santiago* | 19°28'02.21'' | 99°05'23.50'' | 2235 | 03/07 | 24 | 1345 | 37 |
| 28-GM | Centenario* | 19°30'02.52'' | 99°05'44.20'' | 2247 | 03/07 | 32 | 2640 | 39 |
| 29-GM | Dolores San Juanico* | 19°30'17.57'' | 99°05'56.29'' | 2248 | 03/07 | 25 | 1660 | 51 |
| 30-GM | Nezahualcoyotl S. Isabel* | 19°30'00.26'' | 99°06'57.01'' | 2245 | 04/07 | 20 | 1200 | 39 |
| 31-GM | Cartagena San Pedro* | 19°30'17.26'' | 99°07'13.40'' | 2266 | 04/07 | 30 | 2530 | 40 |
| 32-GM | Santa Maria Ticoman* | 19°31'12.35'' | 99°08'00.44'' | 2255 | 04/07 | 20 | 2260 | 41 |
| 33-GM | Santiago Atepetlac* | 19°31'20.24'' | 99°09'41.25'' | 2246 | 04/07 | 35 | 2490 | 50 |
| 34-GM | Cuauhtepec* | 19°33'05.39'' | 99°08'26.84'' | 2286 | 04/07 | 69 | 2930 | 47 |
| **IC** | **Iztacalco** |  |  |  |  |  |  |  |
| 35-IC | San Jose Iztacalco* | 19°23'34.92'' | 99°07'47.75'' | 2236 | 28/03 | 85 | 7300 | 23 |
| **IP** | **Iztapalapa☼** |  |  |  |  |  |  |  |
| 36-IP | De San Jose Aculco* | 19°22'31.89'' | 99°06'08.60'' | 2238 | 28/03 | 25 | 3420 | 31 |
| 37-IP | Civil San Nicolas Tolentino* | 19°20'32.07'' | 99°05'01.17'' | 2296 | 04/09 | 149 | 4765 | 55 |
| 38-IP | Civil San Lorenzo Tezonco** | 19°18'36.10'' | 99°03'50.60'' | 2252 | 04/09 | 150 | 2345 | 44 |
| 39-IP | La Cuevita* | 19°21'24.48'' | 99°05'31.83'' | 2256 | 04/09 | 25 | 1710 | 52 |
| 40-IP | 5 De Mayo-Galeana* | 19°20'27.05'' | 99°02'58.92'' | 2246 | 28/08 | 35 | 1845 | 52 |
| 41-IP | Reforma Municipal* | 19°20'21.72'' | 99°01'38.28'' | 2252 | 28/08 | 35 | 3075 | 39 |
| 42-IP | De Culhuacan* | 19°20'24.82'' | 99°06'24.88'' | 2252 | 28/03 | 20 | 1730 | 29 |
| 43-IP | Mirador Santiago* | 19°21'13.94'' | 99°00'08.08'' | 2273 | 28/08 | 20 | 875 | 49 |
| 44-IP | Santa Martha* | 19°21'47.58'' | 99°00'44.06'' | 2250 | 28/08 | 35 | 2640 | 45 |
| **MC** | **Magdalena Contreras☼** |  |  |  |  |  |  |  |
| 45-MC | San Bernabe* | 19°18'44.54'' | 99°15'21.48'' | 2674 | 31/07 | 92 | 845 | 48 |
| 46-MC | San Francisco* | 19°18'50.21'' | 99°13'58.16'' | 2481 | 31/07 | 35 | 1255 | 49 |
| 47-MC | San Nicolas* | 19°17'51.02'' | 99°14'04.31'' | 2518 | 31/07 | 25 | 995 | 47 |
| 48-MC | San Jeronimo* | 19°19'99.24'' | 99°13'32.90'' | 2417 | 31/07 | 20 | 1485 | 51 |
| **MH** | **Miguel Hidalgo☼** |  |  |  |  |  |  |  |
| 49-MH | Israelita* | 19°24'09.88'' | 99°12'22.87'' | 2347 | 07/08 | 25 | 1320 | 53 |
| 50-MH | Civil De Dolores* | 19°24'04.96'' | 99°12'44.20'' | 2372 | 07/08 | 214 | 4210 | 67 |
| 51-MH | Frances* | 19°27'08.76'' | 99°12'42.74'' | 2274 | 21/08 | 60 | 4800 | 51 |
| 52-MH | Sanctorum* | 19°27'15.81'' | 99°12'54.89'' | 2283 | 21/08 | 60 | 3960 | 52 |
| 53-MH | Espanol* | 19°27'51.13'' | 99°12'36.42'' | 2265 | 14/08 | 230 | 3595 | 47 |
| 54-MH | Americano* | 19°27'33.80'' | 99°12'02.86'' | 2259 | 14/08 | 25 | 1340 | 49 |
| 55-MH | Aleman* | 19°27'38.52'' | 99°11'56.54'' | 2257 | 14/08 | 20 | 915 | 51 |
| 56-MH | Britanico* | 19°27'31.94'' | 99°11'57.20'' | 2257 | 14/08 | 20 | 1005 | 48 |
| 57-MH | Monte Sinai* | 19°27'31.56'' | 99°12'00.79'' | 2259 | 14/08 | 25 | 1150 | 51 |
| **MA** | **Milpa Alta ☼** |  |  |  |  |  |  |  |
| 58-MA | Milpa Alta*** | 19°11'22.14'' | 99°00'49.03'' | 2413 | 10/07 | 25 | 1460 | 52 |
| 59-MA | San Francisco Tecoxpa** | 19°11'41.65'' | 99°00'12.47'' | 2338 | 10/07 | 15 | 1130 | 60 |
| 60-MA | San Jeronimo Miacatlan** | 19°11'26.61'' | 99°00'04.05'' | 2406 | 10/07 | 15 | 950 | 47 |
| 61-MA | San Juan Tepenahuac** | 19°11'23.45'' | 98°59'43.87'' | 2424 | 10/07 | 15 | 1160 | 51 |
| 62-MA | De Santa Ana Tlacotenco** | 19°10'21.45'' | 99°00'07.54'' | 2671 | 10/07 | 30 | 2895 | 49 |
| 63-MA | Salvador Cuauhtenco*** | 19°11'03.33'' | 99°05'37.07'' | 2882 | 10/07 | 25 | 1845 | 48 |
| 64-MA | San Pablo Oztotepec*** | 19°10'32.46'' | 99°04'14.83'' | 2827 | 10/07 | 30 | 2420 | 56 |
| 65-MA | San Lorenzo Tlacoyucan** | 19°10'33.96'' | 99°01'52.79'' | 2623 | 10/07 | 10 | 619 | 51 |
| 66-MA | San Pedro Atocpan** | 19°12'04.38'' | 99°03'16.63'' | 2473 | 11/07 | 25 | 2560 | 39 |
| 67-MA | Bartolome Xicomulco*** | 19°12'04.91'' | 99°04'06.80'' | 2606 | 11/07 | 20 | 1310 | 49 |
| 68-MA | San Antonio Tecomitl*** | 19°12'49.69'' | 98°58'54.60'' | 2246 | 11/07 | 35 | 1725 | 50 |
| **TH** | **Tlahuac☼** |  |  |  |  |  |  |  |
| 69-TH | General San Pedro Tlahuac* | 19°16'05.01'' | 99°00'01.93'' | 2240 | 17/07 | 260 | 3865 | 48 |
| 70-TH | Tierra Blanca* | 19°14'28.09'' | 98°59'54.92'' | 2265 | 17/07 | 25 | 1020 | 38 |
| 71-TH | Lirios*** | 19°13'48.01'' | 98°59'05.02'' | 2243 | 17/07 | 15 | 740 | 46 |
| 72-TH | Tetelco*** | 19°12'34.24'' | 98°58'26.14'' | 2251 | 11/07 | 15 | 940 | 47 |
| 73-TH | San Andres Mixquic* | 19°13'31.64'' | 98°57'49.76'' | 2246 | 17/07 | 45 | 2680 | 43 |
| 74-TH | Tlaltenco San Francisco* | 19°17'57.95'' | 99°01'17.21'' | 2248 | 18/07 | 62 | 1360 | 39 |
| 75-TH | Zapotitlan** | 19°18'07.39'' | 98°01'31.52'' | 2246 | 18/07 | 30 | 1140 | 51 |
| 76-TH | San Rafael Atlixco*** | 19°18'18.20'' | 99°01'35.38'' | 2252 | 18/07 | 15 | 420 | 48 |
| 77-TH | Santa Catarina*** | 19°18'22.60'' | 98°58'32.96'' | 2243 | 18/07 | 20 | 1290 | 49 |
| **TL** | **Tlalpan☼** |  |  |  |  |  |  |  |
| 78-TL | San Marcos* | 19°16'57.40'' | 99°10'10.28'' | 2295 | 11/09 | 35 | 3720 | 49 |
| 79-TL | Santa Ursula Xitle* | 19°16'48.55'' | 99°10'39.86'' | 2324 | 11/09 | 20 | 1100 | 77 |
| 80-TL | Santo Tomas Ajusco*** | 19°12'51.40'' | 99°13'12.80'' | 3057 | 18/09 | 30 | 1450 | 51 |
| 81-TL | San Miguel Ajusco*** | 19°13'21.77'' | 99°11'51.34'' | 2846 | 18/09 | 30 | 1795 | 48 |
| 82-TL | De La Cruz Petlacalco** | 19°13'59.04'' | 99°10'57.84'' | 2749 | 18/09 | 30 | 2130 | 39 |
| 83-TL | San Miguel Xicalco** | 19°14'02.57'' | 99°10'13.37'' | 2717 | 18/09 | 30 | 1690 | 46 |
| 84-TL | San Andres** | 19°14'54.96'' | 99°10'32.40'' | 2541 | 18/09 | 30 | 940 | 42 |
| 85-TL | San Pedro Martir* | 19°15'58.75'' | 99°10'27.77'' | 2372 | 11/09 | 20 | 1260 | 49 |
| 86-TL | De La Fuerzas Armada☼s* | 19°15'21.74'' | 99°09'37.55'' | 2392 | 11/09 | 15 | 830 | 50 |
| **VC** | **Venustiano Carranza** |  |  |  |  |  |  |  |
| 87-VC | Penon De Los Banos* | 19°26'32.44'' | 99°04'50.88'' | 2249 | 12/09 | 35 | 1230 | 62 |
| **XO** | **Xochimilco☼** |  |  |  |  |  |  |  |
| 88-XO | Jilotepec* | 19°15'39.78'' | 99°07'19.21'' | 2257 | 10/09 | 35 | 2695 | 37 |
| 89-XO | Santiago Tepalcatlapan** | 19°15'04.18'' | 99°07'40.33'' | 2271 | 10/09 | 25 | 2130 | 33 |
| 90-XO | Francisco Tlalnepantla*** | 19°11'37.51'' | 99°07'16.72'' | 2690 | 02/10 | 15 | 930 | 40 |
| 91-XO | San Miguel** | 19°12'20.92'' | 99°08'05.44'' | 2609 | 02/10 | 35 | 2590 | 46 |
| 92-XO | Santa Cecilia Tepetlapa*** | 19°12'35.12'' | 99°06'08.33'' | 2523 | 09/10 | 19 | 985 | 63 |
| 93-XO | Montecristo Santa Cruz** | 19°13'09.35'' | 99°07'37.19'' | 2470 | 02/10 | 15 | 1260 | 61 |
| 94-XO | San Andres** | 19°13'47.14'' | 99°06'30.25'' | 2412 | 09/10 | 35 | 2665 | 58 |
| 95-XO | San Lucas** | 19°14'04.46'' | 99°06'37.85'' | 2349 | 09/10 | 35 | 1720 | 42 |
| 96-XO | San Lorenzo Los Olivos** | 19°14'17.90'' | 99°05'58.58'' | 2312 | 25/09 | 14 | 1045 | 71 |
| 97-XO | Sal Lorenzo Atemoaya* | 19°14'38.26'' | 99°05'50.02'' | 2274 | 25/09 | 35 | 3230 | 41 |
| 98-XO | Las Cruces* | 19°14'35.21'' | 99°04'25.41'' | 2262 | 16/10 | 22 | 930 | 36 |
| 99-XO | Santa Cruz*** | 19°14'10.55'' | 99°04'00.69'' | 2344 | 16/10 | 35 | 1045 | 31 |
| 100-XO | Santiaguito** | 19°15'18.56'' | 99°01'15.26'' | 2256 | 16/10 | 35 | 4735 | 67 |
| 101-XO | San Jose* | 19°15'15.14'' | 99°02'09.01'' | 2267 | 16/10 | 20 | 1745 | 33 |
| 102-XO | San Gregorio* | 19°15'15.78'' | 99°02'52.51'' | 2251 | 16/10 | 35 | 2395 | 51 |
| 103-XO | Santa Maria Tepepan* | 19°16'31.40'' | 99°08'22.27'' | 2282 | 17/10 | 30 | 1335 | 47 |
| **Total** | **103 cemeteries** |  |  |  |  | 3955 | 202,148 | 44.7 |

(*) Urban, (**) Suburban, (***) Rural

^☼^Districts
