# Supplementary figures and images for: The distribution of potential West Nile virus vectors, Culex pipiens pipiens and Culex pipiens quinquefasciatus (Diptera: Culicidae), in Mexico City
Source: Parasit Vectors. 2011 May 9;4:70. doi: 10.1186/1756-3305-4-70 (PMC3117809; doi:10.1186/1756-3305-4-70)

## Slide 1
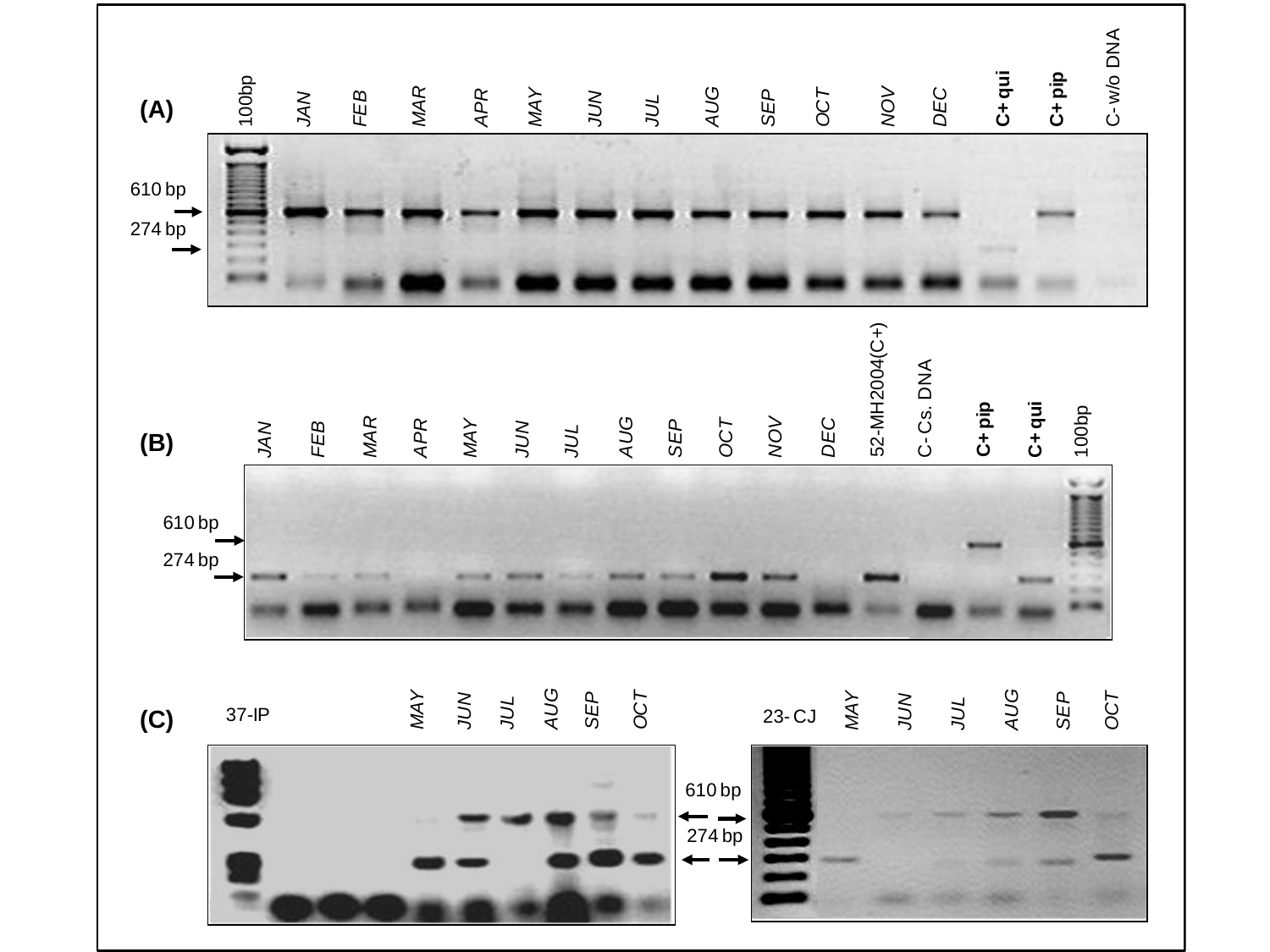

Supplement: Additional File 2 — PCR amplification of the Ace.2 gene from two different cemeteries in 2005. (A) Samples from cemetery "El Calvario" in Cuajimalpa de Morelos borough. All PCR products correspond with the Cx. p. pipiens (610 bp) in each month during 2005. B) Samples from cemetery "Sanctorum" in Miguel Hidalgo borough where the PCR products correspond with Cx. p. quinquefasciatus (274 bp) in each month during 2005. C) Samples from "San Nicolas Tolentino" in Ixtapalapa (37-IP) and from "La Concordia" in Cuajimalp (23-CJ) boroughs where the PCR products correspond with hibryds (610 and 274 bp. Negative controls are indicated as C-Cs.DNA (Culiseta). Positive controls are indicated as C+pip, C+qui and 52-MH2004(C+). 100 bp DNA Ladders are in lines 1 panel A, line 17 panel B and line 1 panel C on the right side; and φ X174 DNA/Hae III ladder on panel C left side. Arrows show the bands of 274 bp and 610 bp in all panels. [file 1756-3305-4-70-S2.PPT]
